# Safety of Accelerated Infliximab Infusions in Children With Inflammatory Bowel Disease: A Retrospective Cohort Study

**DOI:** 10.1097/MPG.0000000000003865

**Published:** 2023-06-15

**Authors:** Jasmijn Z. Jagt, Suzanne E. Galestin, Jürgen Claesen, Marc A. Benninga, Nanne K.H. de Boer, Tim G.J. de Meij

**Affiliations:** From the * Department of Paediatric Gastroenterology and Nutrition, Emma Children’s Hospital, Amsterdam University Medical Centres, University of Amsterdam, Amsterdam, The Netherlands; the † Amsterdam UMC, VU University Amsterdam, Paediatric Gastroenterology, Amsterdam Gastroenterology Endocrinology Metabolism, Amsterdam, Netherlands; the ‡ Faculty of Medicine, Amsterdam UMC, University of Amsterdam, Amsterdam, The Netherlands; the § Department of Epidemiology and Data Science, Amsterdam UMC, Vrije Universiteit Amsterdam, Amsterdam, The Netherlands; the ‖ Department of Gastroenterology and Hepatology, Amsterdam Gastroenterology Endocrinology Metabolism (AGEM) Research Institute, Amsterdam University Medical Centre, VU University Amsterdam, Amsterdam, The Netherlands.

**Keywords:** anti-tumor necrosis factor agents, inflammatory bowel disease, infliximab, infusion reactions, pediatrics

## Abstract

Accelerated infliximab (IFX) infusions have shown to be safe in adults with inflammatory bowel disease (IBD), but data on its safety in pediatric IBD is limited. This study aimed to assess the incidence and timing of infusion reactions (IR) in children with IBD who received accelerated (1-h) versus standard (2-h) IFX infusions.

**Methods:**

This retrospective cohort study included IBD patients 4–18 years of age and initiated IFX between January 2006 and November 2021 at Amsterdam University Medical Centre, location Academic Medical Centre (AMC) and VU Medical Centre (VUmc). The AMC protocol was adjusted in July 2019 from standard to accelerated infusions with 1-h intrahospital post-infusion observation period, whereas in VUmc only standard infusions were administered without an observation period. After merging the departments in 2022, all VUmc patients were allocated to the accelerated infusions (AMC) protocol. Primary outcome was the incidence of acute IR among maintenance accelerated versus standard infusions.

**Results:**

Totally, 297 (150 VUmc, 147 AMC) patients (221 Crohn disease; 65 ulcerative colitis; 11 IBD-unclassified) with cumulative n = 8381 IFX infusions were included. No statistically significant difference in the per-infusion incidence of IR was observed between maintenance standard infusions (26/4383, 0.6% of infusions) and accelerated infusions (9/3117, 0.3%) (*P* = 0.33). Twenty-six of 35 IR (74%) occurred during the infusion, while 9 occurred post-infusion (26%). Only 3 of 9 IR developed in the intrahospital observation period following the switch to accelerated infusions. All post-infusion IR were mild, requiring no intervention or only oral medication.

**Conclusions:**

Accelerated IFX infusion without a post-infusion observation period for children with IBD seems a safe approach.

## Graphical abstract

**Figure:**
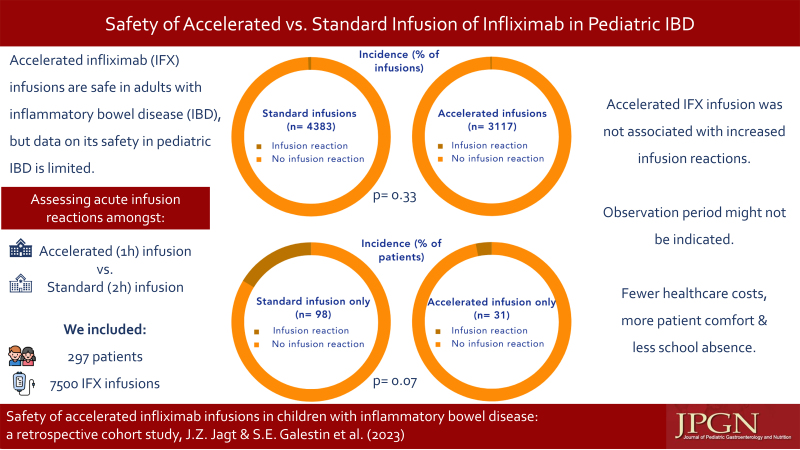

**What Is Known**

- Studies in adults with inflammatory bowel disease (IBD) have shown that accelerated infusion rates of infliximab (IFX) were not associated with an increased risk of infusion reactions.
- The need for an intrahospital observation period following accelerated IFX infusions is still unclear.

**What Is New**

- The administration of accelerated IFX infusions without an intrahospital observation period following infusion seems a safe strategy for all children with IBD receiving IFX.
- The implementation of accelerated IFX infusion without an intrahospital observation period contributes to less school and parental work absenteeism, optimized infusion units capacity, and substantial cost saving.

Infliximab (IFX) is a chimeric monoclonal antibody against tumor necrosis factor-alpha, which is effective for induction and maintenance of remission in children with inflammatory bowel disease (IBD) (1–3). However, the intravenous administration of IFX is associated with infusion reactions (IR). Acute IR are described to occur in 1%–9% of infusions and in 10%–39% of patients (4–6). Although the exact etiology remains unclear, hypotheses include a cytokine release syndrome, anaphylactic reactions, complement activation by anti-IFX antibodies, or degranulation of mast cells and basophils (7). The concomitant use of immunomodulators has been shown to reduce IR risk (8,9), while premedication such as corticosteroids and antihistamines has not been proven effective (6,10).

In current clinical pediatric IBD practices, differences in applied IFX infusion protocols include IFX infusion time and the intrahospital observation period following IFX infusion (11). IFX has been approved to be intravenously administered for at least 2 hours (12), yet accelerated infusion (1 hour) has shown to be equally safe in adults with IBD (13–16). Pediatric data also found no differences in the occurrence of IR between accelerated and standard infusions (17–20). These studies, however, included small numbers of patients who mainly switched to accelerated infusion after tolerating standard infusions (21). Moreover, it remains largely unknown whether an intrahospital observation period after accelerated infusion is indicated. Therefore, this study aimed to assess the incidence of IR and their timing in children with IBD who received accelerated IFX infusions versus standard infusions. Secondary aims were to investigate potential predictive factors for developing acute IR and to evaluate the applied therapeutic strategies in acute IR.

## MATERIALS AND METHODS

### Study Design and Patient Population

In this retrospective cohort study, children 4–18 years of age with IBD (Crohn disease, ulcerative colitis and IBD-unclassified) and initiated IFX in Amsterdam University Medical Centre, locations Academic Medical Centre (AMC) and VU Medical Centre (VUmc), between January 2006 and November 2021, were eligible to participate. Patients were followed until August 2022 or until cessation of IFX. The diagnosis of IBD was based on the revised Porto criteria (22). Exclusion criteria included initiation of IFX at another center and objection to participation.

### Data Collection

The medical records (handwritten and from 2016 electronic) of all patients were reviewed. Patient characteristics were collected, including age, gender, IBD phenotype, disease localization based on the Paris classification (23), and body mass index at start of IFX. The following characteristics of IFX therapy were collected: date of initiation, dosage, concomitant use of immunomodulators, premedication use, anti-IFX antibodies, IFX duration and the number of total IFX infusions, accelerated infusions, and standard infusions. The characteristics of IR included type of symptoms, severity, management, and the timing of the acute IR.

### IFX Infusion Protocol

At both departments, IFX was prescribed according to the European Crohn’s and Colitis Organization (ECCO)-European Society for Pediatric Gastroenterology, Hepatology and Nutrition (ESPGHAN) guidelines (24,25). IFX was administered at 5 mg/kg with 3 induction treatments in week 0-2-6, followed by maintenance treatment every 8 weeks. Both the originator IFX and biosimilar CT-P13 were used. The IFX dose and interval could be adjusted based on therapeutic drug monitoring and disease course. Standard care is to treat all patients with a concomitant immunomodulator including thiopurines and methotrexate, for approximately 6–12 months following the initiation of IFX (24,25).

The 2 departments (AMC and VUmc) of this tertiary IBD center applied different IFX infusion protocols regarding infusion rate and the duration of post-infusion observation period. Since July 2019, the AMC has adjusted their protocol from 2-h IFX infusions to accelerated 1-h infusions with a 1-h intrahospital observation period after infusion, following three 2-h induction infusions. In the VUmc, 2-h standard infusions were administered to patients without an observation period. After merging the departments in June 2022 to location AMC, all VUmc patients switched from standard to accelerated infusions according to the AMC protocol. Consequently, the patients from both departments could be divided into 3 subgroups: (1) patients who exclusively received standard infusions; (2) patients who exclusively received accelerated infusions after three 2-h induction treatments; and (3) patients who switched from standard to accelerated IFX infusions (ie, exposed to both infusion rates).

### Study Outcomes

The primary outcome was the per-infusion incidence of acute IR per maintenance standard versus accelerated infusions. An acute IR was defined as any reaction during infusion or within the 1–2 hours following infusion. Symptoms of acute IR include pruritus, flushing, dyspnea, angioedema, chest discomfort, blood pressure changes (hypo- and hypertension), myalgia, nausea and/or vomiting, rash, headache, and dizziness. Acute IR were graded in severity according to the Common Terminology Criteria of Adverse Events (CTCAE) (26), classified as grade 1 (requiring no intervention), grade 2 (interruption of infusion or therapy required, quick response to therapy), grade 3 (prolonged response to medication or infusion interruption), grade 4 (life-threatening, requiring urgent intervention), and grade 5 (death).

Secondary outcomes were the timing of occurrence of acute IR, potential predictive factors, the management of acute IR, and the incidence of late IR. A late IR was defined as a self-limiting reaction between 1 and 21 days after infusion. Symptoms include skin eruptions, fever, malaise, polyarthralgia, and jaw pain (7).

### Statistical Analysis

Means and standard deviations were calculated for normally distributed data, while medians and interquartile ranges (IQR) were calculated for non-normally distributed data. To assess differences in baseline characteristics between the 2 patient groups (1 and 2), the nonparametric Mann-Whitney *U* test was used for numerical data. Categorical variables were compared using the *χ*^2^ test or Fisher exact test. To investigate an association between acute IR and the administration of accelerated versus standard IFX infusions while accounting for repeated IFX administrations, a generalized binomial model with a random intercept was used. The following potential predictive factors were added to the model: female sex, age at initiation of IFX, the concomitant use of immunomodulators, the presence of anti-IFX antibodies, and premedication use. These factors were considered relevant to the IR risk, based on existing literature and clinical knowledge. Statistical analyses were performed using Statistical Package for the Social Sciences (SPSS, IBM Corp, Armonk, NY, USA; v26) and the statistical software R version 4.0.3 (R Foundation of Statistical Computing, Vienna, Austria). Statistical tests were 2-sided; *P* values <0.05 were considered to be statistically significant.

### Ethics Approval

This study was approved by the medical ethical commission of Amsterdam University Medical Centre on 12th of August 2021, under file number W21_362 # 21.402. Patients and/or their caregivers provided informed consent by opt-out procedure.

## RESULTS

### Baseline Characteristics

**FIGURE 1.**
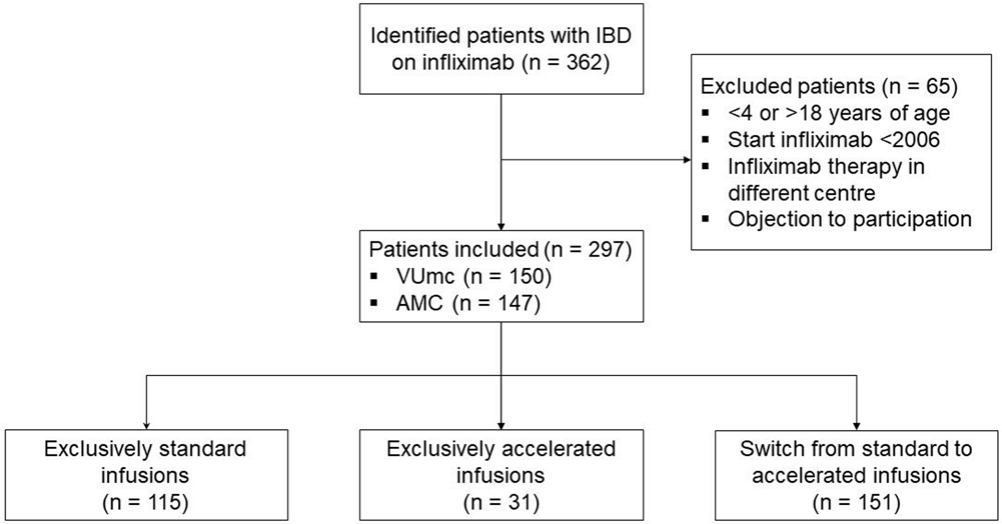
Patient flow chart.

A total of 362 patients with IBD treated with IFX were identified. Of these patients, 65 were excluded due to the following reasons: <4 or >18 years of age when IFX was started (n = 27); initiation of IFX before the year 2006 (n = 4); IFX treatment in another medical center (n = 30); or the patient/caregiver objected to participating (n = 4). Consequently, 297 children were included, of whom 150 and 147 were treated at the VUmc and AMC, respectively. The patient flowchart is shown in Figure 1. A total of 8381 infusions were administered until August 2022, consisting of 5264 standard and 3117 accelerated infusions. One hundred fifteen (38.7%) patients received exclusively standard infusions (group 1), 31 (10.4%) received exclusively accelerated infusions after three 2-h induction infusions (group 2), and 151 (50.8%) switched from standard to accelerated infusions (group 3). The median treatment duration was 31.2 months (IQR 14.1–57.2). The baseline characteristics are depicted in Table 1.

**TABLE 1.** Baseline characteristics

	Exclusively standard infusions	Exclusively accelerated infusions	Switch from standard to accelerated infusions	*P* value*
	(n = 115)	(n = 31)	(n = 151)	
Gender, n (%)				0.016
Female	65 (56.5)	10 (32.3)	71 (47)	
Male	50 (43.5)	21 (67.7)	80 (53)	
Type of IBD, n (%)				0.744
Crohn disease	84 (73)	21 (67.7)	116 (76.8)	
Ulcerative colitis	26 (22.6)	9 (29)	30 (19.9)	
IBD-U	5 (4.3)	1 (3.2)	5 (3.3)	
Age at start IFX in years, median (IQR)	14 (12–16)	16 (14–17)	15 (13–16)	<0.001
BMI at start IFX in kg/m^2^, median (IQR)	18.3 (16.3–20.2)	19.4 (16.2–22.8)	19.1 (16.5–21.1)	0.086
Treatment duration in days, median (IQR)	377 (148–891)	642 (450–895)	1562 (1032–2201)	0.212
Number of maintenance infusions per patient, median (IQR)	7 (2–18)	15 (10–20)	35 (22–48)	0.049
Number of accelerated infusions per patient, median (IQR)	0	15 (10–20)	15 (2–28)	–
Dose in mg/kg, median (IQR)	5.3 (4.9–5.8)	6.1 (5.3–8.8)	5.2 (4.9–5.7)	<0.001
Concomitant IM, n (%)	96 (83.5)	29 (93.5)	142 (94)	0.248
Azathioprine	77 (80.2)	14 (48.3)	111 (78.2)	
Methotrexate	17 (17.7)	14 (48.3)	22 (15.5)	
6-Mercaptopurine	2 (2.1)	1 (3.4)	8 (5.6)	
Thioguanine	0	0	1 (0.7)	
Premedication, n (%)				<0.001
None	44 (38.3)	28 (90.3)	68 (45)	
Hydrocortisone	65 (56.5)	2 (6.5)	82 (54.3)	
Antihistamine	3 (2.6)	1 (3.2)	1 (0.7)	
Other	3 (2.6)	0	0	
Presence of ADA, n (%)	28 (24.3)	3 (9.7)	26 (17.2)	0.087

ADA = anti-drug antibodies; BMI = body mass index; IBD = inflammatory bowel disease; IBD-U = inflammatory bowel disease unclassified; IFX = infliximab; IM = immunomodulator; IQR = interquartile range.

* Baseline characteristics were compared between the group of patients who received exclusively standard versus exclusively accelerated infliximab infusions.

Baseline characteristics were compared between group 1 and group 2. Group 3 was excluded from these analyses. Group 1 consisted of statistically significant more females (*P* = 0.016), younger patients at start of IFX (*P* < 0.001), less maintenance infusions per patient (*P* = 0.049), lower IFX dosage (*P* < 0.001), and more premedication use (*P* < 0.001), compared with group 2. There was no statistically significant difference in the number of patients who used a concomitant immunomodulator or developed anti-IFX antibodies (Table 1).

### Acute IR

#### Induction IFX Infusions

Among 8381 infusions administered to all patients, 47 acute IR were reported (0.6% of total infusions) in 35 of 297 patients (11.8%). Twelve reactions occurred during the induction IFX infusions (26%) in 11 patients. Five reactions developed during the first infusion. The 12 acute IR included grade 1 (n = 5, 42%), grade 2 (n = 5, 42%), grade 3 (n = 1, 8%), and grade 4 IR (n = 1, 8%). The patient with grade 3 IR (during the third infusion) had developed anti-IFX antibodies (290 arbitrary units/mL). Consequently, a switch to adalimumab was performed, which was well tolerated. The grade 4 reaction occurred during the first infusion, followed by a switch to adalimumab. This patient developed antibodies against adalimumab. The remaining 9 patients with grade 1 or 2 IR continued with IFX.

#### Maintenance Accelerated Versus Standard IFX Infusions

For assessing the per-infusion incidence of acute IR among maintenance standard versus accelerated infusions, 881 induction 2-h infusions were excluded. Consequently, 7500 maintenance infusions (4383 standard versus 3117 accelerated infusions) were administered to 280 patients (249 received standard infusions; 182 received accelerated infusions). Thirty-five acute IR (0.5% of total maintenance infusions) occurred in 28 patients (10% of patients).

**FIGURE 2.**
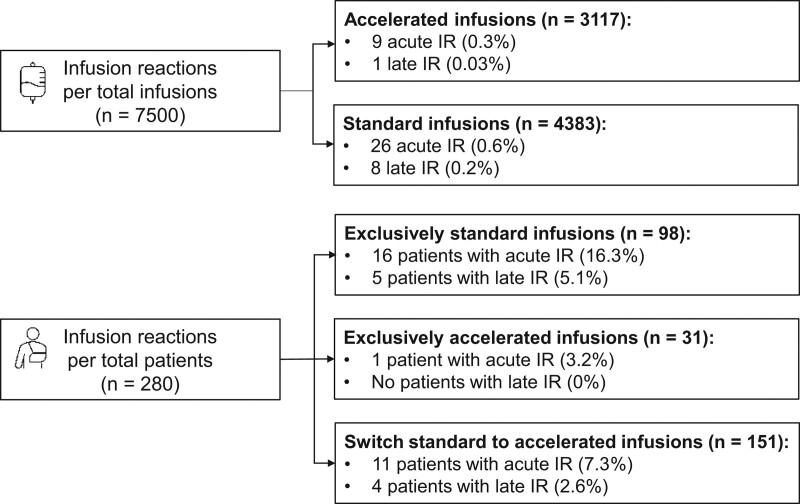
Overview of the incidence of acute and late infusion reactions during the maintenance regimen.

In total, 26 acute IR were reported among 4383 standard IFX infusions (0.6% of infusions) in 20 patients, whereas 9 acute IR occurred among 3117 accelerated infusions (0.3% of infusions) in 9 patients (Fig. 2). The generalized binominal model showed that the administration of accelerated infusions was not associated with the occurrence of acute IR (*P* = 0.334). In the model including the potential predictive factors for IR, the concomitant use of an immunomodulator was associated with a reduced risk of acute IR (incidence risk ratio −2.3, *P* = 0.042), whereas the presence of anti-IFX antibodies was associated with an increased risk of IR (incidence risk ratio 1.7, *P* = 0.043). The use of premedication, gender, and age at the start of IFX were not associated with the risk of acute IR (Table 1, Supplemental Digital Content, http://links.lww.com/MPG/D197).

No significant difference in the number of patients with acute IR was observed between the group receiving exclusively standard infusions (16/98, 16.3% of patients) and the group receiving exclusively accelerated infusions (1/31, 3.2%, *P* = 0.071). In the group of patients who switched from standard to accelerated IFX infusions (n = 151), 14 acute IR occurred in 11 patients (7.3% of patients). Of these 14 acute IR, 6 reactions occurred following standard infusions, whereas 8 reactions developed following the switch to accelerated IFX infusions. The characteristics of all acute IR during the maintenance regimen are depicted in Table 2, including the reported symptoms and the severity based on the CTCAE. One patient developed a grade 4 IR (life-threatening), and no grade 5 IR (adverse event-related death) occurred. Eleven patients had a grade 3 or 4 IR, of whom 8 were tested for anti-IFX antibodies. Seven of these 8 patients (87.5%) had developed anti-IFX antibodies.

**TABLE 2.** Characteristics of acute infusion reactions during maintenance infliximab infusions

	Standard infusion	Accelerated infusion
	(n = 4383)	(n = 3117)
Acute infusion reaction, n (%)	26 (0.6)	9 (0.3)
Severity, n (%)		
Grade 1	6 (0.1)	5 (0.2)
Grade 2	8 (0.2)	3 (0.1)
Grade 3	11 (0.3)	1 (0.03)
Grade 4	1 (0.02)	0
Timing of reaction, n (%)		
During infusion	20 (0.5)	6 (0.2)
After infusion	6 (0.1)	3 (0.1)
Timing infusion reaction* in minutes, mean (SD)	20.6 (11.2)	31 (22.5)
Infusion number†, median (IQR)	7 (6–17)	21 (12–32)
Symptom, n (%)		
Dyspnea	14 (0.3)	1 (0.03)
Flushing	10 (0.2)	1 (0.03)
Nausea and/or vomiting	6 (0.1)	0
Rash	6 (0.1)	3 (0.1)
Headache	6 (0.1)	0
Dizziness	6 (0.1)	4 (0.1)
Angioedema	5 (0.1)	0
Chest pain	2 (0.05)	2 (0.06)
Pruritus	1 (0.02)	1 (0.03)
Hypotension	3 (0.07)	1 (0.03)
Hypertension	0	1 (0.03)
Myalgia	1 (0.02)	0

* Time until infusion reaction occurs, in minutes after starting infusion.

† Infusion sequence number at which the first acute infusion reaction occurred.

#### Timing of Acute IR

The mean timing of occurrence of acute IR was 21 and 31 minutes after starting the infusion in standard versus accelerated infusions, respectively. Among 35 acute IR, 26 IR in 22 patients occurred during the infusion period (74.3%), whereas 9 reactions (25.7%) in 9 unique patients occurred in the post-infusion period. Specifically, 3 IR developed in the intrahospital observation period following accelerated infusions, whereas 6 IR occurred following standard infusions. One patient was at home during the development of the IR. All 9 IR that occurred after the infusion were grade 1 (n = 7) or 2 (n = 2) requiring either no intervention or oral medication such as cetirizine.

### Management of Acute IR

**FIGURE 3.**
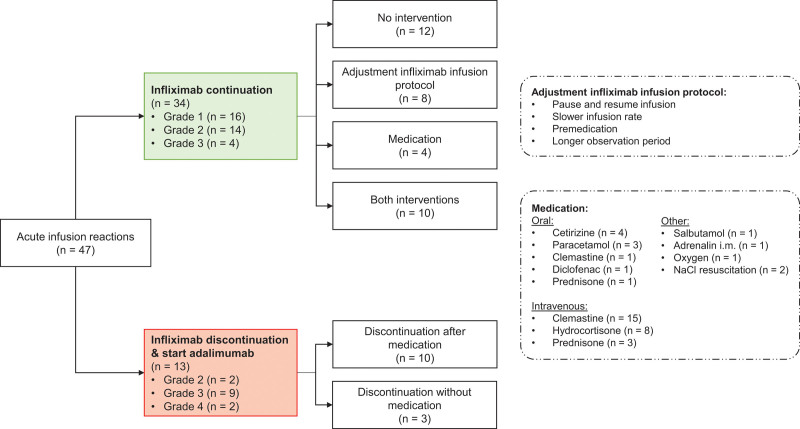
Management of acute infusion reactions.

The applied management of the 47 acute IR that occurred during the induction and maintenance IFX regimen, is summarized in Figure 3. The most severe reactions (grade 4) were treated by repeated intravenous clemastine, intravenous hydrocortisone, fluid resuscitation and, in 1 case, adrenalin as intramuscular injection.

### Late IR

Of all 8381 infusions administered, 15 late IR occurred (0.2% of infusions) in 13 of 297 patients (4.4%). Three of these 13 patients had developed anti-IFX antibodies, assessed at 4 months, 6 weeks, and 1 week before the occurrence of the late IR. Ten of 13 patients (77%) used an immunomodulator next to IFX. When excluding the induction regimen, 9 IR occurred among 7500 maintenance infusions (0.1%) in 9 of 280 patients (3.2%), after a median of the 16th infusion (IQR 10–20). Eight IR developed after standard infusions (8/4383, 0.18%), while 1 IR occurred following accelerated infusions (1/3117, 0.03%). No statistically significant difference was observed in the number of patients with a late IR between the group receiving exclusively standard infusions (5/98, 5.1% of patients) and the group receiving exclusively accelerated infusions (0/31, 0%, *P* = 0.21). Among the patients who switched from standard to accelerated IFX infusions (n = 151), 4 late IR occurred in 4 patients (2.6%). Three late IR developed following standard infusions, whereas 1 developed after an accelerated IFX infusion.

## DISCUSSION

This retrospective study assessed the incidence of acute IR among accelerated (1-h) versus standard (2-h) IFX infusions in children with IBD who were treated at 2 departments of a tertiary center. Accelerated IFX infusions were not associated with an increased risk of acute IR compared with standard IFX infusions. In addition, acute IR that occurred in the post-infusion period were mild (grade 1 or 2). These findings suggest that, following three 2-h induction infusions, IFX could be safely administered in 1 hour without the need for an intrahospital observation period.

To our knowledge, this is the largest study so far assessing the safety of accelerated versus standard IFX infusions in children with IBD. Previous studies consisted of smaller cohorts (16–116 children with IBD) (18–20,27,28) or lacked a control group (17). In addition, multiple studies (19,20) only included children who switched from standard to accelerated infusion, thus acting as their own controls. In the present study, including 297 patients and 8381 IFX infusions, accelerated IFX infusions did not increase the risk of acute IR. This finding supports previous pediatric studies, suggesting that accelerated 1-h IFX infusions are safe (17–20,27,28). A shortened infusion duration of 30 minutes to 1 hour has been studied more extensively in adults with IBD (13–16,29–31). These studies observed that accelerated infusion rates were not associated with an increased risk of acute IR. By implementing 1-h as compared to 2-h infusions in adult IBD patients, Mazzuoli et al (32) showed a significant positive effect on patients’ satisfaction and a reduction of the mean total cost by 47%.

The administration of the accelerated IFX infusions follows the induction regimen consisting of three 2-h IFX infusions. In this cohort, 26% of all acute IR occurred during the induction regimen, including grade 3 and 4 reactions. Five reactions already occurred during the first infusion, which might be caused by a massive cytokine release. It has been hypothesized that the mechanism underlying this cytokine release syndrome, is related to the interaction of the monoclonal antibody IFX with the Fcγ receptor of immune cells, resulting in their activation and the release of cytokines (33). Possible treatment strategies include short-term cessation of IFX infusion, the administration of antihistamines and restarting the infusion at a slower rate (34). This underlines the need for three 2-h induction infusions before switching to accelerated maintenance infusions.

The formation of immune complexes comprising IFX and anti-IFX antibodies, possibly leading to complement activation, has also been proposed as underlying mechanism of IR (7,35). The current data showed that the concomitant use of immunomodulators with IFX was associated with a reduced risk of acute IR, probably by reducing the risk of developing anti-IFX antibodies, as shown in previous adult and pediatric IBD studies (9,36–39). No protective effect of premedication on acute IR was found, which has also been observed in studies including adults and children with IBD who were treated with IFX (6,10).

This study showed that only 3 of 35 acute IR occurred in the 1-h intrahospital observation period following accelerated maintenance infusions, which were all mild (no intervention or oral medication). This suggests that the observation period is not contributing to safety. These results are in line with the prospective study by Lee and colleagues (29) comprising mainly adults with IBD, which showed that IFX infusion can be safely administered over 1 hour without post-infusion monitoring. In a study including patients with psoriasis on IFX, only 1 IR occurred during the post-infusion period among a total of 858 infusions, further supporting the consideration of removing the intrahospital observation period (40). The implementation of accelerated infusions without an intrahospital observation period could lead to decreased school absenteeism, optimized infusion units capacity, and indirect cost savings by minimizing parental work absenteeism.

The strength of this study was the inclusion of patients at 2 departments with different IFX protocols regarding infusion rate, thereby creating an intervention (1-h infusion rate) group and a control (2-h infusion rate) group. In addition, we were able to include a large number of patients with >8000 IFX infusions. The major limitation of this study is its retrospective nature. The assessment of IR relied on the documentation in the medical records by pediatric nurses. This documentation of IR was not performed in a standardized manner, which has resulted in some missing data on the exact timing of IR. Furthermore, delayed, mild IR that occurred outside the hospital might not have been reported by the patients/parents. This could have led to an underestimation of the number of especially delayed IR. Data on IFX dose, premedication, and immunomodulators at initiation of IFX were included as variables in this study. However, adjustments in IFX dose, treatment duration of immunomodulators, and associated therapeutic drug monitoring results were not accounted for. The use of originator IFX or the biosimilar CT-P13 per infusion was not recorded. Therefore, an association between biosimilar use and the occurrence of IR could not be assessed. However, previous studies in children and adults with IBD showed that switching from IFX to biosimilar CT-P13 was not associated with an increase in IR (41,42). The lack of randomization has led to multiple differences in the baseline characteristics between the patient groups, which could have influenced the primary outcome. In addition, there might be a selection bias since developing IR during 2-h infusions could have been a reason not to switch to accelerated 1-h IFX. Likewise, in a few patients with an IR during accelerated infusions, the infusion time was prolonged.

## CONCLUSIONS

In conclusion, this retrospective cohort study showed that accelerated IFX infusion was not associated with an increased incidence of IR compared with standard IFX infusion and thus seems a safe strategy for all children with IBD receiving IFX. Since only mild IR—requiring either no intervention or oral medication—occurred in the intrahospital observation period after infusion, this period might be safely removed. This could lead to less school and work absenteeism and substantial cost savings.

## Supplementary Material



## References

[1] Hyams J Crandall W Kugathasan S. Induction and maintenance infliximab therapy for the treatment of moderate-to-severe Crohn’s disease in children. Gastroenterology 2007;132:863–73; quiz 1165. 17324398 10.1053/j.gastro.2006.12.003

[2] Hyams JS Griffiths A Markowitz J. Safety and efficacy of adalimumab for moderate to severe Crohn’s disease in children. Gastroenterology 2012;143:365–74.e2. 22562021 10.1053/j.gastro.2012.04.046

[3] Hyams J Damaraju L Blank M. Induction and maintenance therapy with infliximab for children with moderate to severe ulcerative colitis. Clin Gastroenterol Hepatol 2012;10:391–9.e1. 22155755 10.1016/j.cgh.2011.11.026

[4] Cheifetz A Smedley M Martin S. The incidence and management of infusion reactions to infliximab: a large center experience. Am J Gastroenterol 2003;98:1315–24. 12818276 10.1111/j.1572-0241.2003.07457.x

[5] Crandall WV Mackner LM. Infusion reactions to infliximab in children and adolescents: frequency, outcome and a predictive model. Aliment Pharmacol Ther 2003;17:75–84. 10.1046/j.1365-2036.2003.01411.x 12492735

[6] van Wassenaer EA Meester VL Kindermann A Koot BGP Benninga MA de Meij TGJ. Premedication with intravenous steroids does not influence the incidence of infusion reactions following infliximab infusions in pediatric inflammatory bowel disease patients—a case-control study. Eur J Clin Pharmacol 2019;75:1445–50. 31332475 10.1007/s00228-019-02715-z

[7] Lichtenstein L Ron Y Kivity S. Infliximab-related infusion reactions: systematic review. J Crohns Colitis 2015;9:806–15. 26092578 10.1093/ecco-jcc/jjv096 PMC4558633

[8] Lichtenstein GR Diamond RH Wagner CL. Clinical trial: benefits and risks of immunomodulators and maintenance infliximab for IBD-subgroup analyses across four randomized trials. Aliment Pharmacol Ther 2009;30:210–26. 19392858 10.1111/j.1365-2036.2009.04027.x

[9] Vermeire S Noman M Van Assche G Baert F D'Haens G Rutgeerts P. Effectiveness of concomitant immunosuppressive therapy in suppressing the formation of antibodies to infliximab in Crohn’s disease. Gut 2007;56:1226–31. 17229796 10.1136/gut.2006.099978 PMC1954977

[10] Jacobstein DA Markowitz JE Kirschner BS. Premedication and infusion reactions with infliximab: results from a pediatric inflammatory bowel disease consortium. Inflamm Bowel Dis 2005;11:442–6. 15867583 10.1097/01.mib.0000158166.88238.ea

[11] Adler J Sandberg KC Shpeen BH. Variation in infliximab administration practices in the treatment of pediatric inflammatory bowel disease. J Pediatr Gastroenterol Nutr 2013;57:35–8. 23459317 10.1097/MPG.0b013e31828f1ea2

[12] US Food and Drug Administration. Drugs@FDA: FDA-Approved Drugs. https://www.accessdata.fda.gov/drugsatfda_docs/label/2021/103772s5401lbl.pdf. Accessed November 20, 2022.

[13] Neef HC Riebschleger MP Adler J. Meta-analysis: rapid infliximab infusions are safe. Aliment Pharmacol Ther 2013;38:365–76. 23815183 10.1111/apt.12389

[14] Clare DF Alexander FC Mike S. Accelerated infliximab infusions are safe and well tolerated in patients with inflammatory bowel disease. Eur J Gastroenterol Hepatol 2009;21:71–5. 19060632 10.1097/MEG.0b013e3283081afe

[15] Ma D Wong W Aviado J Rodriguez C Wu H. Safety and tolerability of accelerated infliximab infusions in patients with inflammatory bowel disease. Am J Gastroenterol 2019;114:352–4. 30333541 10.1038/s41395-018-0368-1

[16] Belhassan M Zeitoun JD Lefevre JH. Infliximab infusion time in patients with inflammatory bowel diseases: is longer really safer? Clin Res Hepatol Gastroenterol 2013;37:189–92. 23246140 10.1016/j.clinre.2012.07.004

[17] El-Matary W Dykes DMH Bauman L. Rapid infliximab infusion in children with inflammatory bowel disease: a multicenter North American experience. Inflamm Bowel Dis 2017;23:2104–8. 29140940 10.1097/MIB.0000000000001259 PMC6551232

[18] Lev-Tzion R Assa A Yerushalmi B. Rapid infliximab infusion in children: a multicenter retrospective cohort study. J Pediatr Gastroenterol Nutr 2017;65:e101–3. 29064928 10.1097/MPG.0000000000001615

[19] Yeckes AR Hoffenberg EJ. Rapid infliximab infusions in pediatric inflammatory bowel disease. J Pediatr Gastroenterol Nutr 2009;49:151–4. 19516188 10.1097/MPG.0b013e31818e1914

[20] Glover C Phuong L Hinds R. Rapid infliximab infusions are generally well-tolerated in children with inflammatory bowel disease. J Paediatr Child Health 2017;53:94–5. 28070959 10.1111/jpc.13384

[21] van Wassenaer EA van Oosterhout JPM Daams JG. Safety of rapid infliximab infusions in children: a systematic review. J Pediatr Gastroenterol Nutr 2020;71:361–5. 32558671 10.1097/MPG.0000000000002815

[22] Levine A Koletzko S Turner D. ESPGHAN revised porto criteria for the diagnosis of inflammatory bowel disease in children and adolescents. J Pediatr Gastroenterol Nutr 2014;58:795–806. 24231644 10.1097/MPG.0000000000000239

[23] Levine A Griffiths A Markowitz J. Pediatric modification of the Montreal classification for inflammatory bowel disease: the Paris classification. Inflamm Bowel Dis 2011;17:1314–21. 21560194 10.1002/ibd.21493

[24] van Rheenen PF Aloi M Assa A. The medical management of paediatric Crohn’s disease: an ECCO-ESPGHAN guideline update. J Crohns Colitis 2021;15:171–94. 10.1093/ecco-jcc/jjaa161 33026087

[25] Turner D Ruemmele FM Orlanski-Meyer E. Management of paediatric ulcerative colitis, part 1: ambulatory care—an evidence-based guideline from European Crohn’s and Colitis Organization and European Society of Paediatric Gastroenterology, Hepatology and Nutrition. J Pediatr Gastroenterol Nutr 2018;67:257–91. 30044357 10.1097/MPG.0000000000002035

[26] In: U.S. Department of Health and Human Services, ed. Common Terminology Criteria for Adverse Events (CTCAE) v5.0; 2017. https://ctep.cancer.gov/protocoldevelopment/electronic_applications/docs/ctcae_v5_quick_reference_5x7.pdf. Accessed July 12, 2022.

[27] Rozette NA Hellauer CM McKee C. Evaluation of rapid vs standard infliximab infusions in the pediatric population. Inflamm Bowel Dis 2018;24:2007–14. 29788416 10.1093/ibd/izy093

[28] O’Connell DM Nachreiner J Shu X. Rapid infliximab biosimilar infusion in children with inflammatory bowel disease. J Pediatr Gastroenterol Nutr 2022;74:605–9. 35149648 10.1097/MPG.0000000000003402

[29] Lee TW Singh R Fedorak RN. A one-hour infusion of infliximab during maintenance therapy is safe and well tolerated: a prospective cohort study. Aliment Pharmacol Ther 2011;34:181–7. 21615434 10.1111/j.1365-2036.2011.04699.x

[30] Babouri A Roblin X Filippi J Hébuterne X Bigard M-A Peyrin-Biroulet L. Tolerability of one hour 10 mg/kg infliximab infusions in inflammatory bowel diseases: a prospective multicenter cohort study. J Crohns Colitis 2014;8:161–5. 23994253 10.1016/j.crohns.2013.08.004

[31] Van Assche G Vermeire S Noman M. Infliximab administered with shortened infusion times in a specialized IBD infusion unit: a prospective cohort study. J Crohns Colitis 2010;4:329–33. 21122522 10.1016/j.crohns.2009.12.012

[32] Mazzuoli S Tricarico D Demma F Furneri G Guglielmi FW. Accelerated infliximab infusion: safety, factors predicting adverse events, patients’ satisfaction and cost analysis. A cohort study in IBD patients. PLoS One 2016;11:e0166443. 27851772 10.1371/journal.pone.0166443 PMC5112916

[33] Vultaggio A Maggi E Matucci A. Immediate adverse reactions to biologicals: from pathogenic mechanisms to prophylactic management. Curr Opin Allergy Clin Immunol 2011;11:262–8. 21460715 10.1097/ACI.0b013e3283464bcd

[34] Maggi E Vultaggio A Matucci A. Acute infusion reactions induced by monoclonal antibody therapy. Expert Rev Clin Immunol 2011;7:55–63. 21162650 10.1586/eci.10.90

[35] van der Laken CJ Voskuyl AE Roos JC. Imaging and serum analysis of immune complex formation of radiolabelled infliximab and anti-infliximab in responders and non-responders to therapy for rheumatoid arthritis. Ann Rheum Dis 2007;66:253–6. 16793840 10.1136/ard.2006.057406 PMC1798492

[36] O’Meara S Nanda KS Moss AC. Antibodies to infliximab and risk of infusion reactions in patients with inflammatory bowel disease: a systematic review and meta-analysis. Inflamm Bowel Dis 2014;20:1–6. 24280879 10.1097/01.MIB.0000436951.80898.6d

[37] Feagan BG McDonald JW Panaccione R. Methotrexate in combination with infliximab is no more effective than infliximab alone in patients with Crohn’s disease. Gastroenterology 2014;146:681–688.e1. 24269926 10.1053/j.gastro.2013.11.024

[38] Church PC Guan J Walters TD. Infliximab maintains durable response and facilitates catch-up growth in luminal pediatric Crohn’s disease. Inflamm Bowel Dis 2014;20:1177–86. 24865777 10.1097/MIB.0000000000000083

[39] Grossi V Lerer T Griffiths A. Concomitant use of immunomodulators affects the durability of infliximab therapy in children with Crohn’s disease. Clin Gastroenterol Hepatol 2015;13:1748–56. 25911120 10.1016/j.cgh.2015.04.010

[40] Wee JS Petrof G Jackson K Barker JNWN Smith CH. Infliximab for the treatment of psoriasis in the U.K.: 9 years’ experience of infusion reactions at a single centre. Br J Dermatol 2012;167:411–6. 22404545 10.1111/j.1365-2133.2012.10931.x

[41] Rusch C Wood M Kennedy AG Tompkins BJ Frasca JD. Rapid infusion of infliximab biosimilars and the incidence and severity of infusion-related reactions in patients with inflammatory bowel disease. J Clin Pharm Ther 2022;47:1851–7. 36134561 10.1111/jcpt.13779 PMC9825869

[42] Gervais L McLean LL Wilson ML. Switching from originator to biosimilar infliximab in paediatric inflammatory bowel disease is feasible and uneventful. J Pediatr Gastroenterol Nutr 2018;67:745–8. 29985877 10.1097/MPG.0000000000002091

