# Supplementary material for: Safety of Accelerated Infliximab Infusions in Children With Inflammatory Bowel Disease: A Retrospective Cohort Study
Source: J Pediatr Gastroenterol Nutr. 2023 Jun 15;77(3):373–80. doi: 10.1097/MPG.0000000000003865 (PMC10417226; doi:10.1097/MPG.0000000000003865)
Supplement: Supplementary file 1 [file mpg-77-373-s001.pdf]

Supplementary Table 1. Generalized binomial model with random intercept of variables associated with the occurrence of acute infusion reactions

	Univariate	
**Variable**	Incidence risk ratio (standard error)	P value
Male sex	-0.84 (0.79)	0.29
Age at start infliximab	-0.12 (0.13)	0.37
Use of IM concomitant with infliximab	-2.29 (1.12)	0.04
Use of premedication	1.31 (0.84)	0.12
Presence of anti-drug antibodies	1.68 (0.83)	0.04
Accelerated infusion rate of IFX	-0.61 (0.55)	0.27

IFX = infliximab, IM = immunomodulator
